# Malignant Ureteral Obstruction: Functional Duration of Metallic versus Polymeric Ureteral Stents

**DOI:** 10.1371/journal.pone.0135566

**Published:** 2015-08-12

**Authors:** Po-Ming Chow, I-Ni Chiang, Chia-Yen Chen, Kuo-How Huang, Jui-Shan Hsu, Shuo-Meng Wang, Yuan-Ju Lee, Hong-Jeng Yu, Yeong-Shiau Pu, Chao-Yuan Huang

**Affiliations:** 1 Department of Urology, National Taiwan University Hospital and National Taiwan University College of Medicine, Taipei, Taiwan; 2 Psychiatric and Neurodevelopmental Genetics Unit and Analytic and Translational Genetics Unit, Massachusetts General Hospital, Boston, Massachusetts, United States of America; 3 Department of Medical Imaging, Cardinal Tien Hospital, School of Medicine Fu-Jen Catholic University, New Taipei City, Taiwan; 4 Department of Medical Imaging, National Taiwan University Hospital and National Taiwan University College of Medicine, Taipei, Taiwan; North Carolina A&T State University, UNITED STATES

## Abstract

**Background:**

Ureteral obstruction caused by extrinsic compression is often associated with intra-abdominal cancers. Internal drainage with ureteral stents is typically the first-line therapy to relieve such obstructions. Novel designs of ureteral stents made of different materials have been invented to achieve better drainage. In this study, we described the functional outcomes of a Resonance metallic ureteral stent (Cook Medical, Bloomington, Indiana, USA) in patients with malignant ureteral obstruction and compare the functional duration of Resonance stents with regular polymeric stents in the same cohort.

**Methods:**

Cancer patients who received polymeric stents and subsequent Resonance stents for ureteral obstruction between July 2009 and November 2012 were included in a chart review. Stent failure was detected by clinical symptoms, imaging studies, and renal function tests. The functional durations of each stent were calculated, and possible factors affecting stent patency were investigated.

**Results:**

A total of 50 stents were successfully inserted into 50 ureteral units in 42 patients with malignant ureteral obstruction. There were 7 antegrade stents and 43 retrograde stents. There were no major complications. Stent-related symptoms were similar in both kinds of stents. After polymeric stents were replaced with Resonance metallic stents, hydronephrosis subsided or remained stable in 90% (45/50) of the ureteral units. Serum creatinine decreased or remained stable in 90% (38/42) of these patients. The Resonance stent exhibited a mean increase in functional duration of 4 months compared with the polymeric stents (p<0.0001), and 50% (25/50) of the Resonance stents exhibited a significant increase in functional duration (more than 3 months). Pre-operative serum creatinine < 2 was associated with a substantial increase in stent duration.

**Conclusions:**

Resonance stents are effective and safe in relieving malignant ureteral obstructions after polymeric stents failure. Resonance stents can provide a longer functional duration than polymeric stents and should be offered as an option for internal drainage.

## Introduction

Ureteral obstruction caused by extrinsic compression is often associated with intra-abdominal cancers. Internal drainage with ureteral stents is typically the first-line therapy to relieve such obstruction. However, the patency rate of regular polymeric stents is low [1, 2]. The median life expectancy of patients with metastatic cancer that causes ureteral obstruction is generally less than one year [3]. Efforts are made to maintain kidney function as well as quality of life. Novel designs of ureteral stents made of different materials have been invented to achieve better drainage. Although segmental metal mesh stents initially seemed promising [4], the long-term results have not been satisfactory [5, 6].

Resonance metallic ureteral stents (Cook Medical, Bloomington, Indiana, USA) possess a unique design. Made of a continuous unfenestrated coil of non-magnetic nickel–cobalt–chromium–molybdenum alloy, the full-length stent has a 1-year indwelling time, according to the manufacturer. The stents have a fixed diameter of 6 Fr., and different length (20–30 cm) are available. The position of the full-length stent is the same as conventional stents; the upper end is situated in the renal pelvis, and the lower end is placed in the urinary bladder.

The use of a Resonance stent was first reported in 2006 in a 64-year-old woman with metastatic breast cancer [7]. Subsequently, a series of studies including both benign and malignant diseases was published [8–18]. Eight of these studies included follow-up results of malignant ureteral obstructions in up to 27 patients [8–15], and 3 studies have analyzed the risk factors of stent failure, including prior radiotherapy, GU cancer, and UTI [12–14]. However, no studies have directly compared the effects of different stents. In this study, we describe our experience of Resonance stents compared with the Universa (Cook Medical, Bloomington, Indiana, USA) polymeric stents in the same cohort. The aim of our study was to estimate the benefit associated with replacing polymeric stents with Resonance stents. The primary outcome was the increase in the functional duration of the stent, and the secondary outcomes were the factors associated with the increase in stent durations.

## Materials and Methods

Ethics Statement: The research meets all applicable standards for the ethics of experimentation and research integrity. The National Taiwan University Hospital Research Ethics Committee and Institutional Review Board approved this retrospective study and waived the informed consent requirement. The IRB case number was 201208052RIC. Patient information was anonymized and de-identified prior to analysis.

In our hospital, the antegrade method of stent insertion was performed under local anesthesia by radiologists, and the retrograde method was performed under general anesthesia by urologists. During the retrograde procedure, initially, an introduction set similar to an 8/10F dilator is passed over a guidewire into the renal pelvis. The wire and inner catheter are removed, and Resonance stent is passed through the 10F introducer sheath. The inner catheter is then used to advance the stent proximally until the pigtail is deployed in the renal pelvis. Finally, the 10F sheath is retracted with the inner catheter held in place until the distal pigtail is deployed in the bladder. The technique is similar with the antegrade method. Prophylatic antibiotics were used for all patients prior to the procedure. Routine follow-up studies include urinalysis, renal function test, plain x-ray, and renal ultrasound. Additional studies such as urine culture, antegrade pyelography, computed tomography (CT), or magnetic resonance imaging (MRI) were performed if clinically indicated.

Cancer patients who received Resonance stents for ureteral obstruction between July 2009 and November 2012 were included in a chart review. Patients with no previous polymeric stents were excluded. No patients were excluded due to missing data, and no patients were lost to follow-up prior to stent failure. Bilateral ureters in the same patient were viewed as individual ureteral units (UUs). Patient profiles and lab data were collected. Stent-related complications were reviewed. A urinary tract infection (UTI) was defined as a positive culture with urinary symptoms. Images prior to and following the procedure were reviewed to confirm the resolution and recurrence of obstruction. The degree of hydronephrosis was measured at least twice: before the placement of polymeric stents and before the placement of metallic stents. Additional imaging studies were available in some cases, which were indicated. In an analysis of risk factors, the highest degree of hydronephrosis ever detected during a follow-up period in the same renal unit was used to represent the degree of obstruction.

The duration of each stent was measured from the day of insertion to the day of removal or to the day when a percutaneous nephrostomy (PCN) was performed. For those who decided not to have their stent removed due to terminal disease status, the date of stent failure was defined as the date progressive hydronephrosis was confirmed by an imaging study. The functional duration of the last polymeric stent and the following metallic stent in each patient were calculated, as was the difference in the durations. The stents were then divided into two groups according to their outcomes: those with good outcomes who had an additional functional duration > 3 months compared with polymeric stents, and those with poor outcomes who had an additional functional duration ≦ 3 months or shorter than polymeric stents. The 3-month cut off ensured that the additional duration did not result from delayed detection of stent failure and that there was a substantial benefit.

We first described the baseline characteristics of the patients, including gender, age, and body mass index (BMI), as well as the number of patients and ureteral units across cancer types. We used Kaplan-Meier curve with 95% confidence intervals (CI) to describe the functional durations of polymeric stents and metallic stents. The difference in stent duration was calculated and tested with a paired-t test. We also described the functional outcomes of the patients after the metallic stents. Finally, we investigated the potential factors that may increase the functional duration of the stents over 3 months. The factors included the following: age ≧ 60 years, gender, method of insertion (antegrade vs. retrograde), previous technical failure, genitourological (GU) cancer, previous and ongoing radiotherapy/chemotherapy, pre-operative serum creatinine < 2 mg/dL, severe hydronephrosis, and occurrence of UTI. Odds ratios were estimated and tested with Fisher’s exact test. Statistical analyses were performed using free software (R version 3.0.1). All statistical tests were two-tailed, and p<0.05 indicated statistical significance.

## Results

From July 2009 to November 2012, 42 cancer patients with malignant ureteral obstruction underwent replacement of their polymeric stents with Resonance stents in our hospital. These patients were followed until the end of January 2014, when all of the metallic stents had malfunctioned. There were 14 (33%) men and 28 (66%) women. The mean±sd age was 57.2±12.3 years old (range: 32–83 years old). The mean±sd body height was 159.5±7.7 cm (range: 142.5–174 cm), and the mean±sd body weight was 56.0±16.6 kg (range: 35.8–115.4 kg). The mean±sd BMI was 21.9±5.6 kg/m^2^ (range: 13.4–42.2 kg/m^2^). Cervical cancer was the most frequent type of malignancy (11 patients), followed by colorectal cancer (9 patients), and gastric cancer (5 patients) (Table 1). There were 13 obstruction sites in the upper ureter, 22 in the middle ureter, and 15 in the lower ureter.

**Table 1:** The number of the patients, ureteral units, and stents according to type of malignancy.

	Patient	Ureteral unit
Anal cancer	1	1
Bladder cancer	3	5
Breast cancer	1	1
Cervical cancer	11	14
Colorectal cancer	9	10
Endometrial cancer	3	3
Gastric cancer	5	6
Lung cancer	1	1
Lymphoma	2	3
Malignant neoplasm of small intestine	1	1
Ovary cancer	3	3
Prostate cancer	2	2

**Fig 1:**
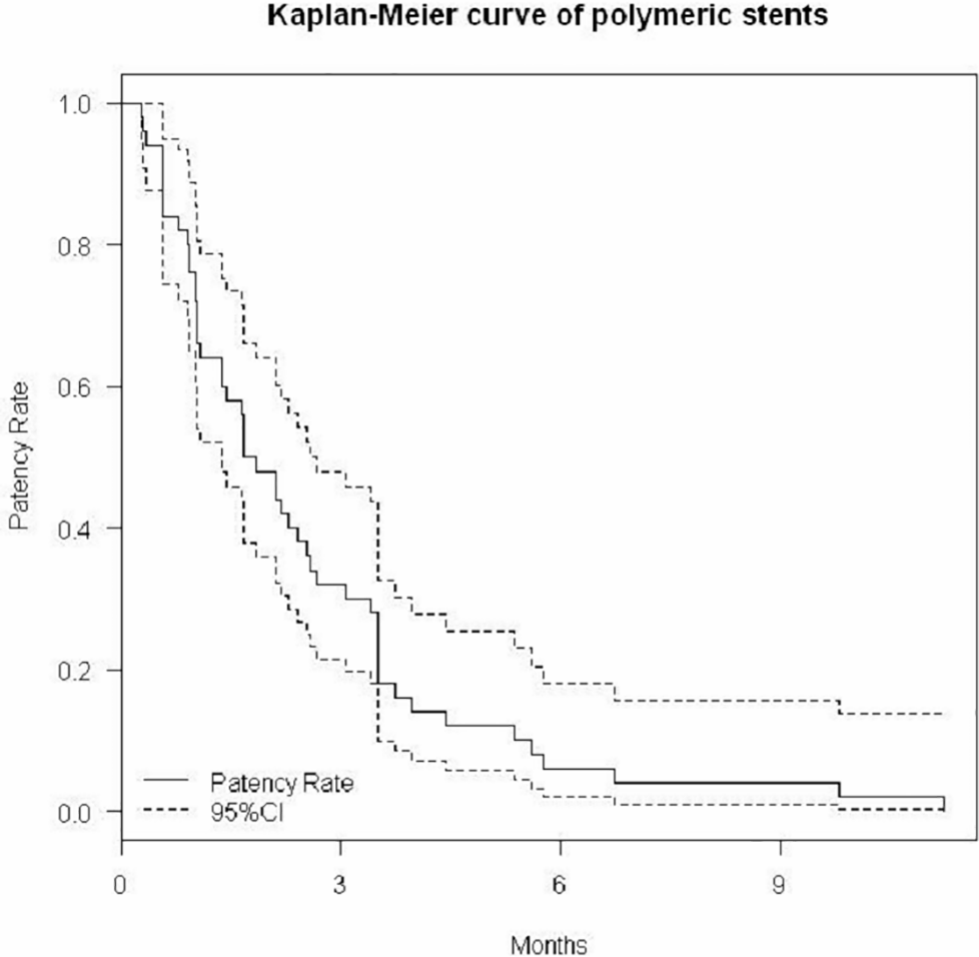
Kaplan-Meier curve of polymeric stents.

**Fig 2:**
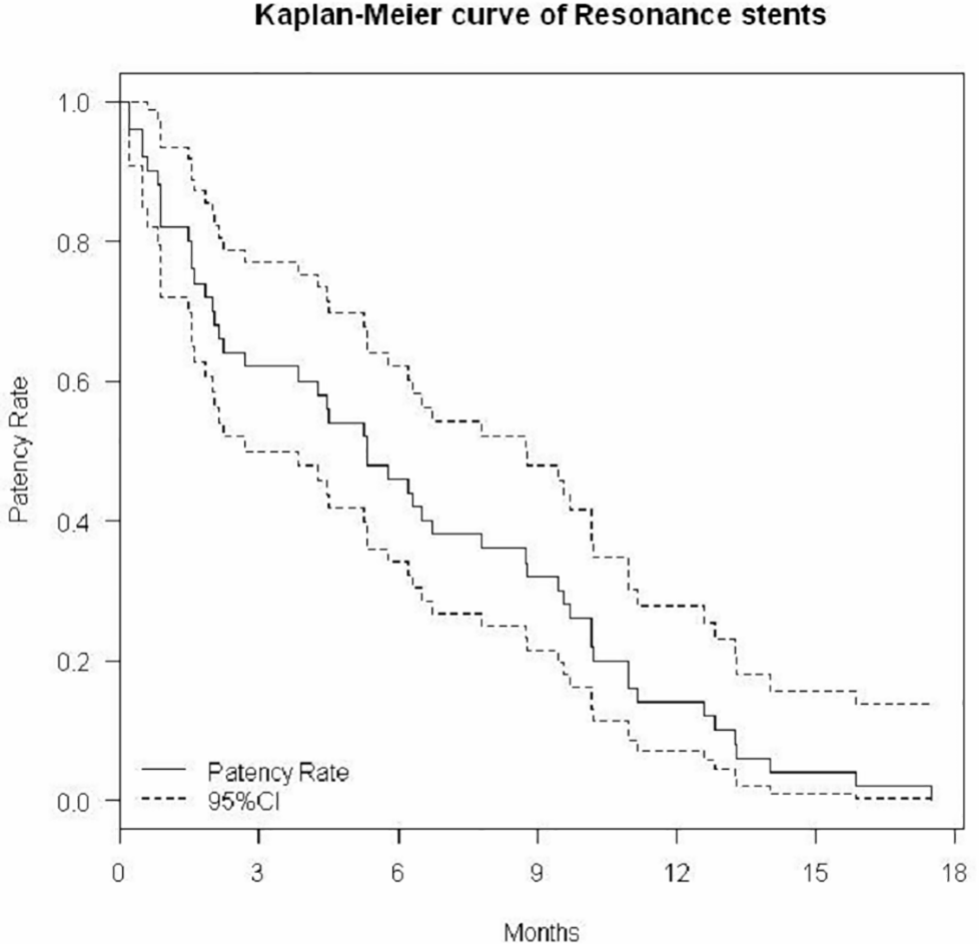
Kaplan-Meier curve of Resonance stents.

A total of 50 stents were successfully inserted into 50 ureteral units in 42 patients. There were 8 patients with bilateral stents. After replacing polymeric stents with Resonance metallic stents, hydronephrosis subsided or remained stable in 90% (45/50) of the ureteral units. Serum creatinine decreased or remained stable in 90% (38/42) of the patients. The median functional durations for the polymeric stents and metallic stents were 1.7 (95%CI: 1.4–2.6) months and 5.3 (95%CI: 2.7–8.8) months, respectively (Fig 1 and Fig 2). The Resonance stent had a mean increase of 4 months in functional duration compared with the polymeric stents (p<0.0001). An increase in functional duration of more than 3 months was observed in 50% (25/50) of the metallic stents (Table 2). There were 1 patient with dysuria, 1 with fever, 4 with urinary frequency, and 5 with hematuria for polymeric stents, and there were 1 patient with dysuria, 3 with fever, 1 with flank pain, 3 with urinary frequency, and 6 with hematuria for metallic stents. There were no stents exchanged due to non-tolerability. These cases were managed conservatively, and no major complications were reported.

**Table 2:** The functional outcomes of the patients and the metallic stents.

**Post-operative serum creatinine**		Decreased	Stable	Increased	Total
Number of patients		28	10	4	42
%		66.67	23.81	9.52	
**Hydronephrosis**	Completely resolved	Improved	Stable	Worse	Total
Ureteral units	16	17	12	5	50
%	32	34	24	10	
**Increase in functional duration**	Decrease	0–3 months	3–6 months	> 6 months	Total
Number of stents	14	11	11	14	50
%	28	22	22	28	

After dividing the stents according to their additional functional durations (> 3 month vs. ≦ 3 months relative to polymeric stents), there were 25 stents in each group. Pre-operative serum creatinine < 2 mg/dL was associated with good stent outcomes (odds ratio = 28.4, p-value = 0.0001). Age ≧ 60 years, gender, method of insertion (antegrade vs. retrograde), previous technical failure, GU cancer, previous and ongoing radiotherapy, severe hydronephrosis, and occurrence of UTI were not significantly associated with good stent outcomes (Table 3).

**Table 3:** The effects of clinical factors on the outcome of stents.

		Duration increase > 3 months		Odds ratio	p-value	
		Yes	No			
Total		25	25			
Age> = 60	Yes	10	14	0.5307	0.3961	
	No	15	11			
Gender	Male	7	9	0.6965	0.7624	
	Female	18	16			
Method of insertion	Antegrade	4	3	1.3875	1.0000	
	Retrograde	21	22			
Previous technical failure	Yes	5	6	0.7954	1.0000	
	No	20	19			
GU cancer	Yes	2	5	0.3550	0.4174	
	No	23	20			
Ongoing radiotherapy	Yes	2	2	1.0000	1.0000	
	No	23	23			
Previous radiotherapy	Yes	13	9	1.9004	0.3931	
	No	12	16			
Ongoing chemotherapy	Yes	10	10	1.0000	1.0000	
	No	15	15			
Previous chemotherapy	Yes	21	18	2.0417	0.3057	
	No	4	7			
Pre-op Cre< 2	Yes	24	11	28.4468	0.0001	*
	No	1	14			
Severe hydronephrosis	Yes	16	20	0.4518	0.3451	
	No	9	5			
UTI	Yes	7	7	1.0000	1.0000	
	No	18	18			

## Discussion

In our study of 50 Resonance stents in 42 patients with malignant ureteral obstruction, the Resonance stents exhibited an average of 4 months of additional functional duration compared with polymeric stents. After polymeric stent failure, the Resonance stents continued to maintain renal function in 90% of the patients. Of all the factors analyzed, pre-operative serum creatinine appeared to be the only significant factor related to a good stent outcome. Based on our result, we recommend placing metallic stents in those with preserved renal functions.

The Resonance stent was designed to sustain stronger extrinsic compressions. In 2006, Borin et al reported the first successful case managed with a Resonance stent in a patient with retroperitoneal fibrosis resulting from metastatic breast cancer. The stent lasted 4 months [7]. In other studies, the median duration ranged from 3.5 to 11 months [9–13], and there were no major complications. The safety and efficacy of the Resonance stent is well-recognized. Although the Resonance stent is expected to provide a longer duration than regular polymeric stents, since the first report in 2006, no studies have directly compared their functional duration, in either a cohort or randomized study. By comparing the sequential functional durations of different stents in each patient, we confirmed the superior efficacy of the Resonance stent.

Three studies assessed risk factors associated with the failure of Resonance stents and reported conflicting results. In a cohort of 19 patients including both malignant and benign obstructions, Wang et al indicated that the proportion of patients with prior radiotherapy was significantly higher in the failure group [12]. On the contrary, in an analysis of 25 patients with malignant ureteral obstruction, Goldsmith et al found that radiation was not associated with stent failure. The authors also found that GU cancer and tumor invasion to the bladder were predictors of stent failure [13]. Brown et al. observed more post-operative UTI in the obstruction group than in the patent group [14]. In our study, neither radiotherapy, GU cancer, nor UTI were associated with a reduction of stent duration.

In 3 studies using polymeric stents, various factors for stent failure were investigated. Yossepowitch et al studied 39 patients with extrinsic ureteral obstruction from both benign and malignant diseases, and multivariate logistic regression revealed that the degree of hydronephrosis was a significant predictor of stent failure [19]. Ganatra et al described a retrospective cohort of 157 patients with malignant extrinsic ureteral compression, indicating that invasion at cystoscopy exhibited significant predictive value for progression to PCN [20]. Jeong et al described another retrospective cohort of 86 patients with non-urological malignancies and did not identify any predictors for stent failure in a univariate analysis [21]. In our study, severe hydronephrosis did not affect the improved stent duration of Resonance stents, which implies that the Resonance stents were more effective in relieving severe obstruction.

Our results indicated that pre-operative creatinine was associated with stent duration. The relationship between high pre-operative serum creatinine and stent failure might be inadequate urine production, which could more easily cause encrustation on the stents and thus affect stent duration. Liatsikos et al observed encrustation in 12 out of 54 stents in benign and malignant patients, but the effect of encrustation on stent duration was not analyzed [10]. Due to insufficient information about stent encrustation from the medical records, we were also unable to analyze stent encrustation in our study. However, in patients with malignant ureteral obstruction, higher serum creatinine might be related to more advanced disease and more rapid cancer progression, both of which might affect stent durations. Patients with higher serum creatinine levels may also exhibit more co-morbidities and be more susceptible to UTI, thus prompting stent replacement or external drainage sooner than in patients with lower serum creatinine levels. These possible explanations are only hypotheses. Further studies are required to investigate the association between serum creatinine and stent duration.

The advantage of the Resonance stent is mainly based on its long-term durability. Frequent replacement of the stent could be avoided, thereby improving patient quality of life and reducing medical costs [17,22]. Overall, stent-related symptoms were similar in both kinds of stents in our study, probably resulting from the super-elastic design of the Resonance stent. Although the effect of Resonance stents on quality of life might vary between individuals, there are no studies using validated questionnaires to assess subjective tolerability. Based on our results, the effect on medical costs in any health care system can be estimated from the 4-month extension of functional duration.

There were several sources of biases in our study. First, although we used the same cohort as the control, an ideal study would be to place both stents at the time of initial diagnosis and compare stent durability. Other than a randomized control study, placing two kinds of stents into the same ureter would create identical environment for both stents. However, it would be difficult to measure the patency of the polymeric stent and metallic stent respectively. If additional space created between two stents provides extra drainage or prolongs duration remains unclear. The effect of combining two different stents has not been reported and might be of interest in a future study.

With limited case number, we tried to eliminate potential confounders by using the patients as their own control. Other than our study design, there are alternative ways to compare the duration of the stents. A standard cohort study requires one group of patients with metallic stents and another group with polymeric stents, ether after prior polymeric stents or as their initial management. This method would result in comparing different stents in different patients, in which potential confounders might exist without proper randomization. Another possible study design is to use metallic stents first and then replace them with polymeric stents. However, the duration of polymeric stents following metallic stents were almost always shorter in our experience. The shorter duration could be ether the effect of less sustainable stent design or simply the result of disease progression, which would be a confounder that is difficult to measure or control.

The dynamics of the cancer status was another source of bias. Although the cancer status likely did not change significantly during our short period of follow-up, it was indeed dynamic due to the nature of the cancer and the treatment course. The conventional follow-up interval with cross-sectional imaging for malignancy is 3 months, which is usually longer than the functional duration of polymeric stents (median 1.7 months). Therefore, we could not effectively detect whether the tumors progressed or regressed during the placement of polymeric or metallic stents. Nonetheless, we can assume that if the tumors progressed, they would be larger when the metallic stents were indwelling, making the durability of the stents more significant. We do not know whether the increased stent duration resulted from tumor shrinkage, which could be an effect of additional treatment. To compensate for the lack of assessment for cancer dynamics, we included both prior and ongoing use of chemotherapy and radiotherapy in the risk analysis, and the results were insignificant.

In addition to the concern of cancer status, the major challenge in studies of ureteral stents in malignant ureteral obstruction is the measuring a true effect of tumor compression on ureters. Although the tumor bulk can be measured with cross-sectional images, the correlation between tumor bulk and compression force is uncertain. A balloon catheter connecting to a pressure transducer might be useful to measure the precise intraluminal pressure, but there is no in vivo data from clinical settings.

Compared with other studies, there are several advantages of our study: a relatively large number of patients receiving Resonance stents, a homogenous cohort consisting only of cancer patients, the sequential use of different stents in the same cohort, thereby minimizing inter-group differences in a non-randomized setting. However, our study does have some limitations. First, this was a retrospective study with no strict follow-up protocol. Second, considering the progressive disease status in patients with malignant ureteral obstruction, the benefit of metallic stents in our cohort might be underestimated. Third, without the use of diuretic renography, which is largely limited by the local health reimbursement system, split renal function could not be accurately evaluated. Finally, the data are insufficient for cost and quality-of-life analyses. A prospective randomized study is needed to more accurately assess the difference in the function durations of the stents. Future studies can use a prospective protocol to identify more factors that might influence stent duration, and these factors can be examined in patients receiving other types of stents.

## Conclusion

Resonance stents are effective and safe in relieving malignant ureteral obstructions after the failure of polymeric stents. The stents can provide a longer functional duration than polymeric stents, and should be offered as an option for internal drainage.

## Supporting Information

S1 Table Duration difference of each stent. Dura diff: duration difference
(XLS)
